# Functional, Structural and Genetic Modulation in Plasma and Renal Antioxidant Systems by Quercetin, Catechin and Genistein in Cisplatin‐Induced Acute Nephrotoxicity in Wistar Rats

**DOI:** 10.1002/fsn3.70680

**Published:** 2025-08-11

**Authors:** Pawan K. Verma, Priyanka Sharma, Sheen Tukra, Bhawani Singh, Shilpa Sood, Nrip K. Pankaj, Biswajit Brahma, Gholamreza Abdi, Zuhaib F. Bhat

**Affiliations:** ^1^ Division of Veterinary Pharmacology and Toxicology F.V.Sc., SKUAST‐Jammu, J&K Jammu India; ^2^ Division of Veterinary Pathology F.V.Sc., SKUAST‐Jammu, J&K Jammu India; ^3^ Division of Livestock Production and Management F.V.Sc., SKUAST‐Jammu, J&K Jammu India; ^4^ Department of Biotechnology, Persian Gulf Research Institute Persian Gulf University Bushehr Iran; ^5^ Division of Livestock Products Technology SKUAST‐Jammu, J&K Jammu India

**Keywords:** catechin, cisplatin, genistein, glutamate‐cysteine ligase, nephrotoxicity, quercetin

## Abstract

The study aimed at gaining mechanistic insights into the modulation of cisplatin‐induced (cDDP, 12 mg/kg‐IP) renal toxicity by quercetin, catechin, and genistein in Wistar rats. Blood was analyzed for alterations in acute renal biomarkers (KIM‐1, Cystatin‐C, GGT, BUN, CR, UA) together with the changes in the activities of antioxidant biomarkers (TAS, TTH, GSH) and cellular damage indicators (MDA, AOPP and 8‐OHdG). Additionally, alterations in the activities, variations in genetic expression of antioxidant enzymes (CAT, SOD, GPx, GST, AE, GR) and histomorphological lesions in the kidneys were studied. Quercetin, catechin, and genistein restored the renal antioxidant status and thiol levels, highlighting their potential in conferring protection against cDDP‐induced damage to renal parenchyma as evidenced by the reduction of lesion load in the affected kidneys. Results demonstrated their capabilities to modulate the activities of GCLC, GCLM, effector thiols, and other components of antioxidant machinery to offset cDDP‐induced nephrotoxicity. Furthermore, it can be gleaned from our data that quercetin proved to be more effective in alleviating cDDP‐induced kidney damage in comparison to catechin and genistein.

## Graphical abstract

Cisplatin administration increases production of reactive oxygen species (ROS) as indicated by increased plasma renal biomarkers (KIM‐1, Cys‐C, BUN, CR), cellular damage indicators (MDA, AOPP, 8‐OHdG) and histopathology of kidney. Co‐exposure along with quercetin, catechin and genistein restored the renal functions which may be due to direct ROS scavenging potential, increased renal GR activity and GCL expression leading to increased cellular thiol (‐SH) production.

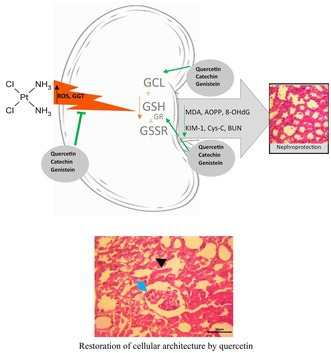

## 1 Introduction

Cisplatin (cDDP) is considered an extremely effective anticancer treatment for cancers of the ovary, breast, stomach, lung, head, and neck, but this anticancer therapy is known to inflict numerous side effects such as oxidative stress mediated damage to the kidney, liver, and heart (Baba et al. 2016; Elsayed et al. 2021, 2022). However, nephrotoxicity is the major side effect of cDDP therapy and is directly proportional to the dose escalation required to attain optimal therapeutic levels, which limits its clinical usage (Elsayed et al. 2021; Fang et al. 2021). Prolonged retention of cDDP and its toxic metabolites in renal tubular epithelial cells causes loss of brush borders, epithelial cell necrosis, sloughing, and cast formation to cause renal obstruction followed by infiltration of lymphocytes and macrophages at the site of injury (Eljack et al. 2014; Fang et al. 2021). Diminution of cDDP‐induced nephrotoxicity will remove hurdles for its long‐term use or its employability in treatment regimens necessitating its heavy dose usage to harness the maximal antineoplastic potential. Antioxidative enzymes, particularly thiols (–SH group), play multifaceted roles in maintaining vital physiological processes essential for cellular homeostasis (Singh, Verma, et al. 2020; Verma et al. 2016, 2018). However, the alterations in redox brought about by the unbalanced free radicals or reactive intermediaries in drug‐induced nephrotoxicity can deplete body thiol reserves. The existing body of epidemiological and experimental studies indicates that restoration of thiols bolsters the antioxidant status and minimizes drug‐induced organ injury (Sharma et al. 2023; Verma et al. 2016, 2018). The nuclear factor erythroid 2‐related factor 2 (Nrf_2_) directly regulates mitochondrial ROS homeostasis by detoxification of peroxides, regeneration of thiols such as reduced glutathione (GSH), and activities of other endogenous antioxidant enzymes, which might be differentially regulated in a variety of human disorders (Sheng et al. 2023; Shirvanian et al. 2024; Walencik et al. 2024; Zhang et al. 2024). Moreover, Nrf_2_ is an important regulator of glutamate‐cysteine ligase (GCL, which consists of GCLC and GCLM subunits) which catalyzes the rate‐limiting step in the synthesis of GSH. The stimulation of thiols is a crucial event that shields against oxidative stress, dampens the damage to essential cellular macromolecules, tissue architecture, and also boosts the activities of other antioxidants.

Over the recent years, researchers around the world have been meticulously screening and evaluating the potential of different phytochemicals in the management of cDDP‐induced renal injury (Eugenio‐Pérez et al. 2016; Fang et al. 2021). Phytochemicals like quercetin, catechin, and genistein possess immense potential to serve as natural antioxidants for combating drug‐induced cellular damage. Quercetin and catechin are widely studied bioflavonoids, abundantly present in foods including broccoli, pepper, coriander, citrus fruits, apples, berries, red onions, grapes, cherries, and tea. Many studies have documented quercetin's remarkable counteractive potential against cDDP‐induced toxicity. Quercetin‐enriched floral extract prepared from *Calendula officinalis* mitigated cDDP renal toxicity (Verma et al. 2016). Najafi et al. (2022) have also reviewed the role of quercetin as an adjunct polychemotherapeutic to reduce harmful side effects of cDDP, and it has been supported by evidence from other in vitro as well as xenograft mouse studies (Li et al. 2019). Quercetin alleviated cDDP‐induced oxidative damage to hepatic parenchyma (Verma et al. 2018). Similarly, isoflavonoid genistein, a principal constituent in soybean and its derivatives and other legumes, is a phytoestrogen associated with health benefits largely owing to its strong anti‐inflammatory and antioxidant properties (Goh et al. 2022). Moreover, quercetin, catechin, and genistein are excellent chelators of metal ions such as Cu^2+^ and Fe^2+^ and can possibly stem the barrage of oxidant onslaught by intermediates of cDDP metabolism formed in renal epithelial cells. Furthermore, being rich in flavanols, these compounds are seasoned exogenous scavengers of oxidizing moieties.

We wanted to delineate functional and molecular modalities exercised by quercetin, catechin, and genistein in preventing oxidative damage to the kidney as a result of cDDP administration in Wistar rats. Hence, the present research work was conducted to determine whether quercetin, catechin, and genistein supplementation can affect activities and expression not only of endogenous thiols and other components of antioxidant machinery but also of upstream factors involved in their replenishment such as Nrf_2_ as well as GCL (GCLM and GCLC) to prevent cDDP‐induced nephrotoxicity. Also, the abilities of the three natural compounds to confer protection to the renal histoarchitecture and to ameliorate changes in clinical markers of kidney injury altered by cDDP toxicity were evaluated.

## 2 Materials and Methods

### 2.1 Experimental Animals and Ethical Statement

In vivo experimental studies were conducted on adult Wistar rats of either sex weighing 180–200 g obtained from the Indian Institute of Integrative Medicine (IIIM), Jammu. The animals were provided standard management conditions (22°C ± 3°C, 50%–60% relative humidity, and 12 h dark and light cycle), fed pelleted ration, and provided clean drinking water *ad libitum*. The rats were acclimatized in the laboratory conditions for a period of more than 2 weeks before the start of the experiment. All the experimental animals were kept under constant observation during the entire period of study for the development of any toxicological clinical signs. The experiments were conducted according to established ethical guidelines (CPCSEA, India) and the study complied with all the regulations. The experimental protocols were duly approved by the Institutional Animal Ethics Committee (IAEC, registered with CCSEA No. 25/341/2010‐AWD) vide proposal no 10/IAEC‐20/2020. The animals received humane care following the National Institute of Health Guide for the Care and Use of Laboratory Animals (NIH Publication No. 85‐23, revised 1996).

### 2.2 Experimental Design

Thirty apparently healthy Wistar rats of either sex were randomly allocated into five groups with six rats in each. Group I served as control; group II animals were administered cisplatin (cDDP) at the dose of 12 mg/kg b. wt. intra‐peritoneally (IP). Group III, IV, and V animals were pre‐exposed to quercetin (100 mg/kg b. wt. IP), catechin (100 mg/kg b. wt. IP), and genistein (15 mg/kg b. wt. IP), respectively, 1 h before cDDP administration and also at 24 and 48 h after the exposure to cDDP (Verma et al. 2016, 2019). At the end of the experiment, that is, on the 4th day, blood samples were collected directly from the heart, and animals were sacrificed by cervical dislocation. Renal tissue (1 g) was collected in 10 mL ice‐cold 0.5 M phosphate buffer (pH 7.4) for the estimation of antioxidant parameters. Renal tissue homogenate (10%) was prepared by homogenizing the tissue using a Teflon‐coated homogenizer (IKA‐T10, ULTRA‐TURRAX, Germany) at 1000 rpm for 5–7 min at 4°C. Renal tissues for histopathological studies were collected in 10% formal saline, and for mRNA expression (RNAiso plus, TaKaRa, Japan) studies, tissues were collected in TRIzol.

### 2.3 Plasma Biochemical Parameters

Different renal biomarkers viz. blood urea nitrogen (BUN), creatinine (CR), uric acid (UA), total plasma proteins (TPP), and albumin (ALB) were estimated in plasma by standard kits (Transasia Bio‐Medicals Ltd., India) with the help of a Chemistry Analyzer (CHEM‐7, ERBA, Manheim). The levels of kidney injury molecule‐1 (KIM‐1), cystatin‐C (Cys‐C), and interleukin‐18 (IL‐18) were estimated using ELISA kits supplied by FineTest Wuhan Fine Biotechnology, China. The absorbance of ELISA plates was recorded by an ELISA microplate reader (EPOCH2, BioTek, USA).

### 2.4 Oxidative Stress Biomarkers

Estimation of various antioxidant biomarkers viz. catalase (CAT), superoxide dismutase (SOD), glutathione peroxidase (GPx), glutathione reductase (GR), glutathione S transferase (GST), arylesterase (AE), reduced glutathione (GSH), nitric oxide (NO), acetylcholinesterase (AChE) and total antioxidant status (TAS), total thiols (TTH) were carried out in erythrocyte lysate and renal tissue homogenate as per the methods previously described using UV–Visible spectrophotometer (Evolution One, Thermo Scientific, US) [CAT (Aebi 1974), SOD (Marklund and Marklund 1974), GPx (Hafeman et al. 1974), GR (Carlberg and Mannervik 1985), GST (Habig et al. 1974) and AChE (Voss and Sachsse 1970), TTH (Mochnik et al. 1994), TAS (Re et al. 1999), AE (Furlong et al. 1988), NO (Sastry et al. 2002), GSH (Beutler 1975)].

### 2.5 Cellular Damage Indicator

Malondialdehyde (MDA) and advanced oxidation protein products (AOPP) were determined using standard methods of Shafiq‐Ur‐Rehman (1984) and Witko‐Sarsat et al. (1996), respectively. The extent of DNA damage was determined by estimating the levels of 8‐hydroxy‐2′‐deoxyguanosine (8‐OHdG) in the plasma of control and treatment groups using ELISA kits (FineTest, Wuhan Fine Biotechnology, China) using an ELISA microplate reader (EPOCH2, BioTek, USA).

### 2.6 Primer Designing for Expression Analysis of Genes by qRT‐PCR

The primers for real‐time PCR amplification of the above‐mentioned genes were designed by analyzing ESTs available in the NCBI GenBank database. The sequences were retrieved using the BLAST tool of NCBI. Multiple sequence alignments were performed in CLUSTAL Omega. The exon boundaries were analyzed in the UCSC genome browser using rat genome assembly (Oar_v4.0/oviAri4). The forward and reverse primers were picked from conserved exon fragments flanking intron(s). The specificity of the primers was tested *in silico* using the BLAST tool of NCBI and the BLAT tool of UCSC. The list of the primers used for real‐time PCR amplification of the above‐mentioned genes is provided in Table 1.

**TABLE 1:** The list of primers used for real‐time PCR amplification of different antioxidant genes.

Batch #	Oligo name	Oligo #	Sequence (5′–3′)
BA01573912	RATACHERTFW	3007191004‐130/0	ATCGAGTTCATCTTTGGGCTCCCCCTGGA
BA01573913	RATACHERTRV	3007191004‐140/0	CAGGTTCAGGCTCACGTATTGCTGCGC
BA01573914	RATCATARTFW	3007191004‐150/0	GGAAACAACACCCCTATTTTCTTCATCAGGG
BA01573915	RATCATARTRV	3007191004‐160/0	AGGCAAGTTTTTGATGCCCTGGTCAGTCT
BA01573916	RATGCLCRTFW	3007191004‐170/0	ATCCTCCAGTTCCTGCACATCTACCACG
BA01573917	RATGCLCRTRV	3007191004‐180/0	TCTCCCCCTTCTCTTGCAGAGTTTCAAGAA
BA01573918	RAT GPX4RTFW	3007191004‐190/0	CTGTGGAAATGGATGAAAGTCCAGCCCAAG
BA01573919	RAT GPX4RTRV	3007191004‐200/0	AGATAGCACGGCAGGTCCTTCTCTATCACC
BA01573920	RATGSSRRTFW	3007191004‐210/0	CGTGATGAAGATGGTTTGTGCCAACAAAGAGGA
BA01573921	RATGSSRRTRV	3007191004‐220/0	AGTGTGACCAGCTCTTCTGAAGAGGTAGG
BA01573922	RATGSTA1RTFW	3007191004‐230/0	TCTATGGGAAGGACATGAAGGAGAGAGCC
BA01573923	RATGSTA1RTRV	3007191004‐240/0	CGAGATAATCTTGTCCATGGCTCTTCAACACCT
BA01573924	RATNOSRTFW	3007191004‐250/0	GATGTCACTATGGCAACCAGCGTCCTGC
BA01573925	RATNOSRTRV	3007191004‐260/0	AAAATGTCCTCGTGGTAGCGTTGCTGATCC
BA01573926	RATPON1RTFW	3007191004‐270/0	GCTGACCCATACTTACGGTCCTGGGA
BA01573927	RATPON1RTRV	3007191004‐280/0	GCATGCTTTTCATACACGTGAATCTTGTGAGCC
BA01573928	RATNFE2RTFW	3007191004‐290/0	CAAGAGCAACTCCAGAAGGAACAGGAGAAG
BA01573929	RATNFE2RTRV	3007191004‐300/0	CAAATGGGAATGTCTCTGCCAAAAGCTGCAT
BA01573930	RATSOD1RTFW	3007191004‐310/0	AGGCATGTTGGAGACCTGGGCAATGTG
BA01573931	RATSOD1RTRV	3007191004‐320/0	ACTTTCTTCATTTCCACCTTTGCCCAAGTCATC
BA01573932	RATGCLMRTFW	3007191004‐330/0	CTTTCCTTGGAGCATTTGCAGCCTTACTGG
BA01573933	RATGCLMRTRV	3007191004‐340/0	GCAGTCAAATCTGGTGGCATCACACAGC
BA01573934	RATRPS18RTFW	3007191004‐350/0	TGCGAGTACTCAACACCAACATCGATGGG
BA01573935	RATRPS18RTRV	3007191004‐360/0	CCTCTTGGTGAGGTCAATGTCTGCTTTC

### 2.7 RNA Isolation From Tissue Samples

Total RNA from cells was isolated in TRIzol using the standard method. Briefly, collected tissues were minced with a sterile BP blade, and about 10 mg of minced tissue was taken in 500 μL of TRIzol. Quality check of RNA was performed by agarose (1.5%) gel electrophoresis, and quantity was measured in a spectrophotometer. The RNA isolated was preserved at −20°C till further use.

### 2.8 cDNA Preparation

The total RNAs were converted to cDNA using a commercially available cDNA synthesis kit (RevertAid First Strand cDNA Synthesis Kit, Thermo Fisher Scientific Inc., PA, USA) following the manufacturer's instructions. Briefly, the reaction mixture was prepared in a microfuge tube placed on ice with the following ingredients: total RNA (5 μg), random hexamer primer (50 ng), and DEPC‐treated water (7 μL). The sample mixture was mixed gently, spun for 3–5 min, and incubated at 70°C for 5 min in a thermocycler (Bio‐Rad Laboratories, USA). The tube was then placed on ice, and the following components were added in the indicated order: 5× Reaction Buffer (4 μL), RNase inhibitor (40 U), and dNTPs (10 mM). The components were mixed gently and spun down, and the mixture was incubated at 25°C for 15 min. MMuLV Reverse Transcriptase (200 U/μL) was added, and the mixture was incubated at 25°C for 10 min, followed by at 42°C for 60 min and finally at 70°C for 10 min to stop the reaction. The cDNA prepared was preserved at −20°C till further use.

### 2.9 qRT‐PCR for mRNA Expression Analysis

All qRT‐PCR reactions were conducted on a CFX Connect Real‐Time PCR machine (Bio‐Rad Laboratories, USA). Each reaction consisted of 2 μL cDNA template, 5 μL of 2× SYBR Green PCR Master Mix (iTaq Universal SYBR Green Supermix, Bio‐Rad Laboratories, USA) 0.25 μL each of forward and reverse primers (10.0 pmol/μL) and nuclease‐free water to make a final volume of 10.0 μL. Each sample was run in duplicate as technical replicates. Amplification cycles were programmed as per the manufacturer's instructions and consisted of the following steps: 10 min of initial denaturation at 95°C followed by two segment amplification steps: 15 s at 95°C for denaturation, and 30 s at 72°C for annealing and elongation for 40 × cycles. The melt curve analysis was done with the following cycling parameters: heating from 65°C to 95°C with a 0.5°C increment, 10 s dwell time, and a plate read at each temperature. Raw C_t_ values of samples were analyzed by ΔC_t_ method using housekeeping genes as normalizers and the control group as calibrators.

### 2.10 Histopathological Studies

The histopathological studies on collected renal samples were carried out as per the standard method. The formalin‐fixed renal tissue samples were washed, dehydrated, cleared, embedded in paraffin, sectioned (5 μm), stained with hematoxylin and eosin (H&E), and examined under a light microscope at 400× magnification for histopathological assessment.

### 2.11 Statistical Analysis

The plasma and kidney antioxidant biomarkers were presented as mean ± standard error. The data was analyzed by one‐way analysis of variance (ANOVA) followed by Tukey's post hoc test to statistically analyze the data, and all values were presented as the mean ± standard error of the mean. Statistical differences were considered significant for *p* < 0.05 (IBM, statistics SPSS 21.0).

## 3 Results

### 3.1 Blood

**FIGURE 1:**
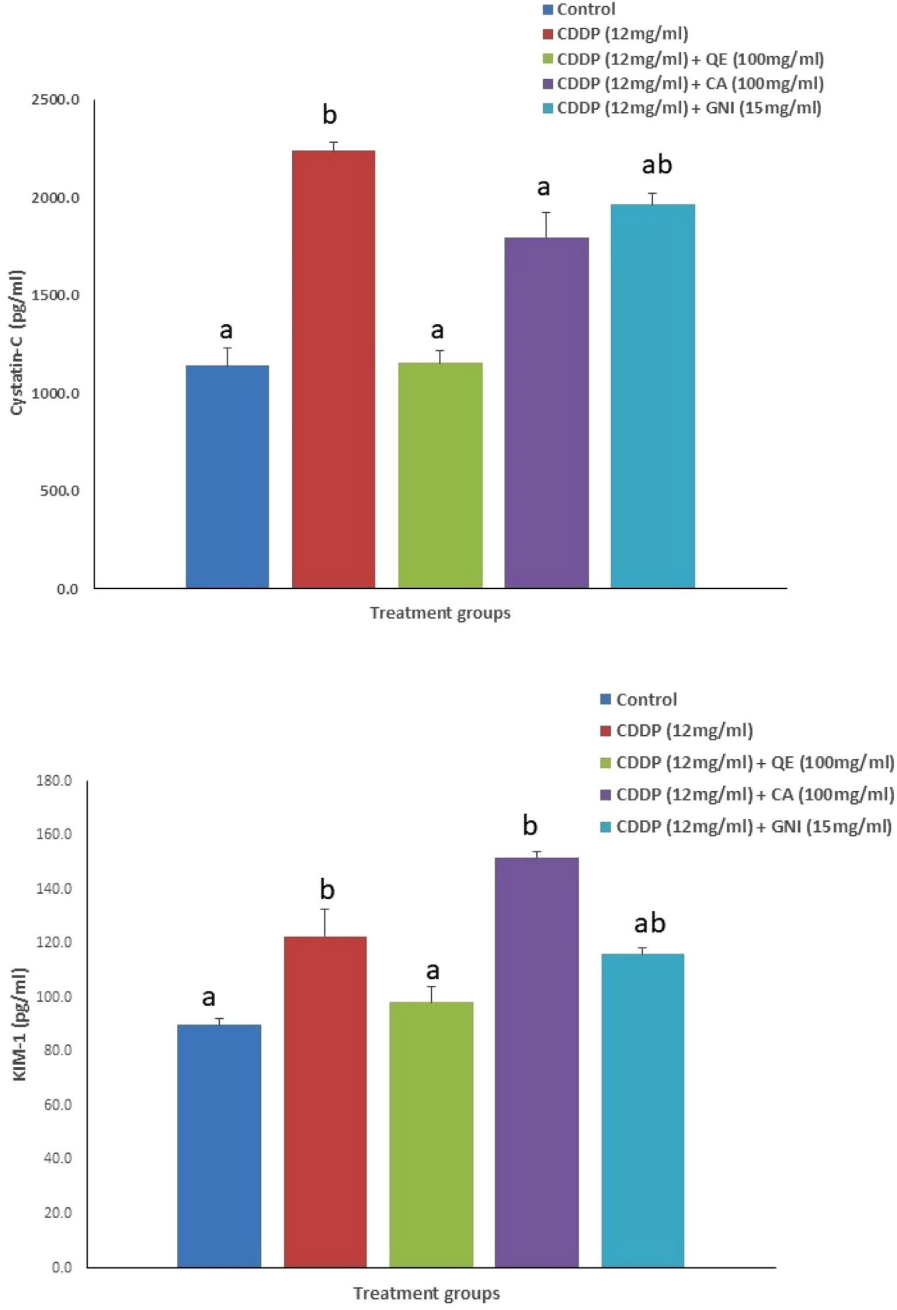
Plasma levels of Kidney Injury Molecule‐1 (KIM‐1) and cystatin‐C in control and treated Wistar rats (CA, catechin; cDDP, cisplatin; GNI, genistein; QE, quercetin).

The effect of cDDP alone and in combination with quercetin, catechin, and genistein on plasma biomarkers indicative of renal health is depicted in Table 2. Total protein and albumin levels were significantly reduced in rats exposed to cDDP. All three phytoingredients counteracted the effect of cDDP, but not significantly. The levels of BUN, CR, and UA showed significant elevation after cDDP treatment; however, quercetin and catechin, but not genistein, completely restored CR and UA levels, even though BUN was only partially corrected by all three preventive agents. Levels of KIM‐1 and Cystatin‐C were significantly increased after the cDDP administration. Quercetin significantly reduced the levels of both, while significant improvements after genistein supplementation were seen only in the levels of KIM‐1. Co‐treatment with catechin didn't cause any significant alteration in the levels of any of the renal injury biomarkers (Figure 1).

**TABLE 2:** Ameliorative efficacy of phytoingredients (quercetin, catechin, and genistein) on plasma renal biomarkers in cDDP‐induced nephrotoxicity in Wistar rats.

Groups	TPP (gm/dL)	ALB (gm/dL)	BUN (mg/dL)	CR (mg/dL)	UA (mg/dL)
Control	6.53^b^ ± 0.43	3.86^b^ ± 0.25	22.29^a^ ± 1.45	0.70^a^ ± 0.02	10.05^a^ ± 0.45
Cisplatin (cDDP)	4.28^a^ ± 0.42	3.18^a^ ± 0.18	42.37^d^ ± 1.52	1.12^b^ ± 0.08	17.74^b^ ± 0.83
cDDP + quercetin	5.23^ab^ ± 0.29	3.72^ab^ ± 0.30	36.49^c^ ± 1.72	0.80^a^ ± 0.04	12.45^a^ ± 0.38
cDDP + catechin	4.89^ab^ ± 0.30	3.67^ab^ ± 0.13	31.92^b^ ± 1.41	0.82^a^ ± 0.08	12.29^a^ ± 0.69
cDDP + genistein	4.43^a^ ± 0.27	3.61^a^ ± 0.25	34.24^bc^ ± 0.92	0.77^a^ ± 0.03	15.79^b^ ± 1.59

*Note:* Values are given as mean ± SE of 6 animals unless otherwise stated. Values having different superscripts (a, b, c) in a column are statistically different from one another at a 5% level of significance.

Abbreviations: ALB, albumin; BUN, blood urea nitrogen; CR, creatinine; TPP, total plasma proteins; UA, uric acid.

Plasma GSH, TTH, GR, GPx, and GST levels were significantly decreased, and GGT levels were significantly raised after cDDP administration. The administrations of these phytoingredients were equally and completely effective in rebounding GSH, TTH, and GR. GPx and GGT levels were fully recovered by genistein, but only partial improvements were witnessed in GPx as well as GGT levels after quercetin and catechin supplementation. While genistein did not affect GST, it could be completely restored by quercetin and catechin (Table 3). The administration of cDDP caused significant depletion of antioxidant enzymes (CAT, SOD, AE, AChE, and TAS) in the blood. Complete recovery was recorded in the levels of all these antioxidants after co‐treatment with all three ameliorative phytoingredients, the exception being genistein, which could not cause any change in the fallen AE levels (Table 4).

**TABLE 3:** Ameliorative efficacy of phytoingredients (quercetin, catechin, and genistein) on blood thiol homeostasis in cDDP‐induced toxicity in Wistar rats.

Groups	GSH	TTH	GR	GPx	GST	GGT
Control	3.40 ^b^ ± 0.09	0.21^b^ ± 0.03	3.45^b^ ± 0.38	5.39^c^ ± 0.24	1.23^b^ ± 0.09	1.22^a^ ± 0.21
Cisplatin (cDDP)	2.27^a^ ± 0.49	0.14^a^ ± 0.06	2.73^a^ ± 0.42	1.49^a^ ± 0.32	0.88^a^ ± 0.06	2.29^c^ ± 0.35
cDDP + quercetin	3.06^b^ ± 0.35	0.19^b^ ± 0.07	3.14^b^ ± 0.45	3.59^b^ ± 0.53	0.97^b^ ± 0.11	1.96^bc^ ± 0.23
cDDP + catechin	3.11^b^ ± 0.57	0.18^b^ ± 0.02	3.17^b^ ± 0.24	3.61^b^ ± 0.54	0.93^ab^ ± 0.12	1.68^abc^ ± 0.11
cDDP + genistein	3.19^b^ ± 0.55	0.19^b^ ± 0.03	3.09^ba^ ± 0.41	4.20^bc^ ± 0.64	0.91^a^ ± 0.13	1.48^ab^ ± 0.15

*Note:* Values are given as mean ± SE of 6 animals unless otherwise stated. Values having different superscripts (a, b, c) in a column are statistically different from one another at a 5% level of significance. Values of reduced glutathione (GSH) are expressed in mM. Values of TTH (Total thiols) expressed in μM. Values of GR (glutathione reductase) are expressed nmol of NADPH/min. Values of GST (glutathione S transferase) are expressed in μmol of CDNB conjugate formed/min/mg Hb. Values of GPx (glutathione peroxidase) are expressed in Units/mg of Hb. Activities of gamma‐glutamyl transferase (GGT) were expressed in U/L.

**TABLE 4:** Ameliorative efficacy of quercetin, catechin, and genistein on erythrocytes antioxidant system in cDDP‐induced toxicity in Wistar rats.

Groups	CAT	SOD	AE	AChE	TAS
Control	53.09^b^ ± 5.88	50.63^b^ ± 1.86	1.19^b^ ± 0.15	11421.13^b^ ± 389.11	5.56^b^ ± 0.52
Cisplatin (cDDP)	35.45^a^ ± 3.23	36.32^a^ ± 1.94	0.83^a^ ± 0.11	8044.63^a^ ± 376.38	3.89^a^ ± 0.96
cDDP + quercetin	59.27^b^ ± 4.28	49.63^b^ ± 2.22	1.05^b^ ± 0.21	10940.13^b^ ± 304.47	5.34^b^ ± 0.64
cDDP + catechin	46.81^b^ ± 2.11	47.08^b^ ± 5.16	1.02^b^ ± 0.13	11314.75^b^ ± 734.13	5.35^b^ ± 1.12
cDDP + genistein	47.42^b^ ± 3.58	48.12^b^ ± 2.48	0.89^a^ ± 0.13	11187.38^b^ ± 501.73	5.49^b^ ± 0.23

*Note:* Values are given as mean ± SE of 6 animals unless otherwise stated. Values having different superscripts (a, b, c) in a column are statistically different from one another at a 5% level of significance. Values of CAT (Catalase) are expressed in μmol H_2_O_2_ decomposed/min/mg of Hb. Values of SOD (Superoxide dismutase) and GPx (glutathione peroxidase) are expressed in Units/mg of Hb. Activities of arylesterase (AE) expressed in U/mL. Acetylcholinesterase (AChE) activity expressed in nmol of thiol produced/min. Values of TAS (Total antioxidant status), expressed in mM.

**FIGURE 2:**
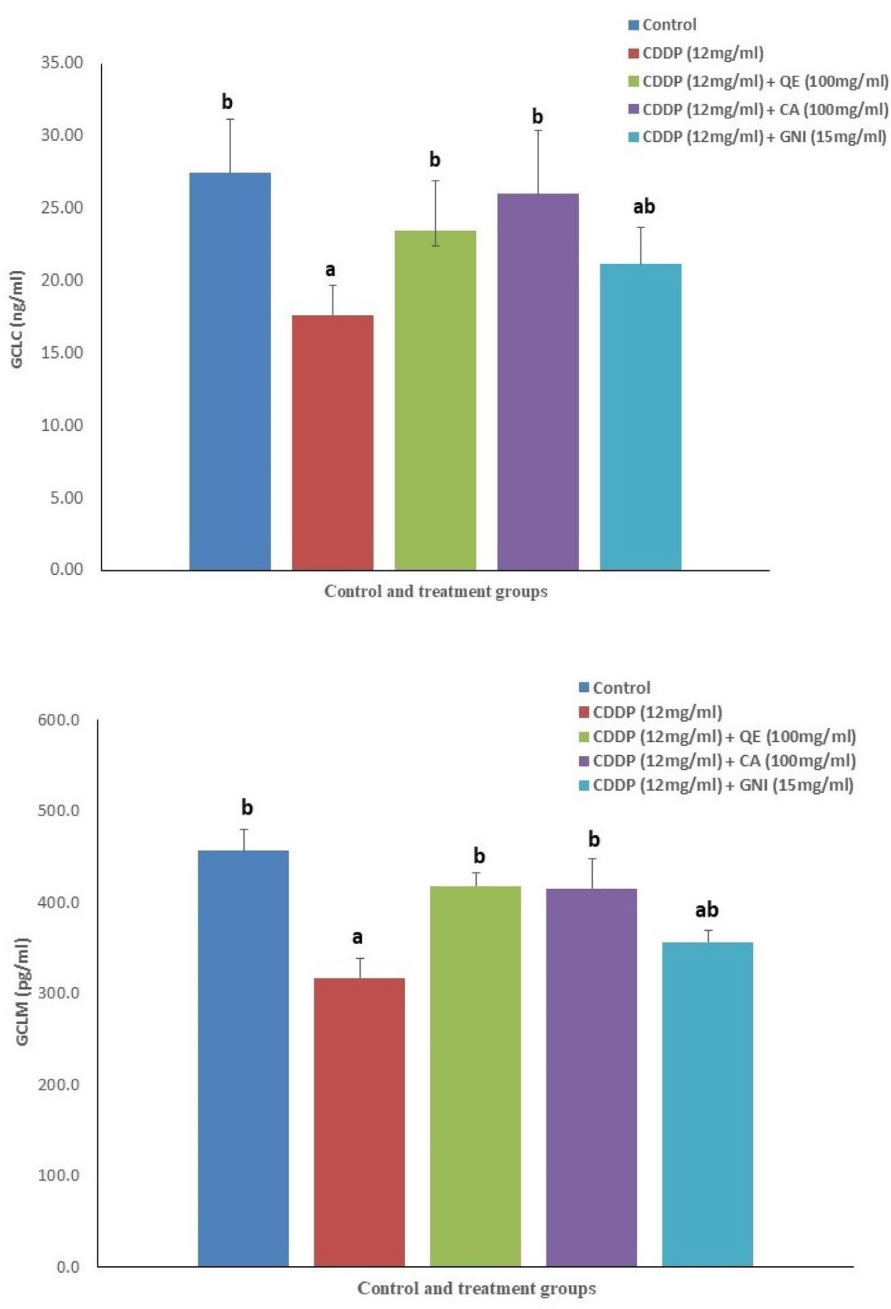
Activity levels of glutamate‐cysteine ligase (GCL) enzyme subunits viz. glutamate‐cysteine ligase catalytic subunit (GCLC) and glutamate‐cysteine ligase modulatory subunit (GCLM) in control and treated Wistar rats (CA, catechin; cDDP, cisplatin; GNI, genistein; QE, quercetin).

The levels of MDA, AOPP, 8‐OHdG, and LI‐18 in plasma were significantly increased after the cDDP administration in rats. Complete amelioration occurred in the levels of MDA and AOPP after the administration of all three preventive agents. Even though complete recovery in 8‐OHdG level occurred after quercetin co‐treatment and substantial but incomplete improvements after simultaneous catechin were noticed, genistein failed to impact the effect of cDDP on 8‐OHdG. A reduced level of IL‐18 was recorded only after catechin administration, whereas quercetin or genistein were unable to bring down IL‐18 levels. (Table 4). The levels of GCLM as well as GCLC concentrations in plasma were significantly reduced after cDDP treatment, but quercetin and catechin completely ameliorated these alterations. On the contrary, genistein could not impact GCLM at all; however, it was able to alter GCLC, but was less powerful than the other two in doing so (Figure 2).

### 3.2 Renal Tissue

Alterations in renal thiols across different experimental groups are depicted in Table 5. Following cDDP administration, levels of GSH, TTH, and activities of GR, GPx, and GST were significantly lower than the values of control rats. Co‐treatment with quercetin, catechin, or genistein led to complete restoration of levels of GSH, TTH, and activities of GR, GPx, and GST in renal tissue. Likewise, a significant depletion in the contents of antioxidant biomarkers such as CAT, SOD, AE, AChE, and TAS was recorded after cDDP administration when contrasted with the respective levels in control animals. After simultaneous treatment with any of the preventive agents, a complete resurrection in the levels of all the measured antioxidant biomarkers was noted, except CAT and AChE levels after genistein and quercetin co‐treatment, respectively, which could not undo the reduction in response to cDDP treatment (Table 6). A significant uptick was recorded in MDA and AOPP levels in the kidneys of group II animals; whereas, concurrent treatment with quercetin, catechin, as well as genistein caused a significant and complete alleviation of MDA and AOPP values, as can be comprehended from Table 7.

**TABLE 5:** Ameliorative potential of quercetin, catechin, and genistein on oxidation of plasma lipids, proteins, nucleic acids, and level of nitric oxide (NO) induced by cDDP in Wistar rats.

Groups	MDA	AOPP	8‐OHdG	IL‐18	NO
Control	1.27^a^ ± 0.17	0.84^b^ ± 0.05	21.81^a^ ± 8.66	74.60^b^ ± 15.21	129.10^c^ ± 12.54
Cisplatin (cDDP)	3.72^b^ ± 0.47	1.34^c^ ± 0.16	372.99^c^ ± 83.84	228.20^c^ ± 41.47	54.92^b^ ± 8.07
cDDP + quercetin	1.49^a^ ± 0.21	0.79^ab^ ± 0.12	65.93^a^ ± 37.25	317.31^ab^ ± 51.26	20.80^a^ ± 3.43
cDDP + catechin	1.61^a^ ± 0.39	0.55^ab^ ± 0.05	102.68^b^ ± 28.78	127.85^ab^ ± 25.26	158.86^c^ ± 11.94
cDDP + genistein	1.43^a^ ± 0.19	0.47^a^ ± 0.04	505.40^c^ ± 74.39	303.44^a^ ± 70.72	15.53^a^ ± 1.84

*Note:* Values are given as mean ± SE of 6 animals unless otherwise stated. Values having different superscripts (a, b, c) in a column are statistically different from one another at 5% level of significance. Values of MDA (malondialdehyde) level are expressed in nmoles MDA produced/mg of Hb/h. Values of advance oxidation protein product (AOPP) are expressed in μM of Chloramine‐T. Values of 8‐hydroxy‐d‐oxyguanosine (8‐OHdG) was expressed in ng/mL. Values of interleukin‐18 (IL‐18) was expressed in pg/mL. Values of nitric oxide (NO) is expressed in μM.

**TABLE 6:** Ameliorative efficacy of quercetin, catechin, and genistein on renal tissue thiol homeostasis in cDDP‐induced nephrotoxicity in Wistar rats.

Groups	GSH	TTH	TAS	GR	GPx	GST
Control	1.33^b^ ± 0.32	2.15^b^ ± 0.21	6.51^b^ ± 1.08	54.48^b^ ± 5.93	180.39^b^ ± 15.24	46.75^b^ ± 5.94
Cisplatin (cDDP)	0.89^a^ ± 0.26	1.49^a^ ± 0.30	4.13^a^ ± 0.50	39.00^a^ ± 8.03	144.96^a^ ± 5.18	29.75^a^ ± 3.91
cDDP + quercetin	1.11^b^ ± 0.22	1.77^ab^ ± 0.27	5.68^ab^ ± 0.89	51.36^b^ ± 5.56	157.58^ab^ ± 10.82	44.78^b^ ± 4.76
cDDP + catechin	1.17^b^ ± 0.32	1.64^ab^ ± 0.44	6.22^b^ ± 0.79	43.68^b^ ± 3.88	166.66^ab^ ± 14.55	40.63^b^ ± 3.03
cDDP + genistein	1.16^b^ ± 0.26	1.77^ab^ ± 0.42	5.75^b^ ± 0.66	49.20^b^ ± 5.19	164.79^ab^ ± 8.38	42.50^b^ ± 4.81

*Note:* Values are given as mean ± SE of 6 animals unless otherwise stated. Values having different superscripts (a, b, c) in a column are statistically different from one another at a 5% level of significance. Values of reduced glutathione (GSH) are expressed in mM. Values of TTH (Total thiols) expressed in μM. Values of TAS (Total antioxidant status), expressed in mM. Values of GR (glutathione reductase) are expressed nmol of NADPH/min. GPx (glutathione peroxidase) are expressed in units/g of tissue. Values of GST (glutathione S transferase) are expressed in μmol of CDNB conjugate formed/min/g of tissue.

**TABLE 7:** Ameliorative efficacy of quercetin, catechin, and genistein on antioxidant biomarkers and cellular damage indicators in renal tissues of Wistar rats.

Groups	CAT	SOD	AE	AChE	MDA	AOPP
Control	6541.06^b^ ± 832.95	1216.46^b^ ± 28.22	1.89^b^ ± 0.37	15537.38^b^ ± 300.70	46.37^b^ ± 7.29	1.52^b^ ± 0.08
Cisplatin (cDDP)	4959.34^a^ ± 260.28	937.81^a^ ± 60.89	1.21^a^ ± 0.17	11699.63^a^ ± 692.86	63.35^a^ ± 2.73	1.76^c^ ± 0.04
cDDP + quercetin	6463.82^b^ ± 377.46	1092.78^b^ ± 27.49	1.41^ab^ ± 0.13	12313.38^a^ ± 257.34	48.75^b^ ± 3.40	1.17^a^ ± 0.02
cDDP + catechin	5404.49^ab^ ± 574.79	1030.13^ab^ ± 38.40	1.52^b^ ± 0.21	14668.63^b^ ± 457.56	57.81^ab^ ± 5.27	1.43^ab^ ± 0.04
cDDP + genistein	6105.23^a^ ± 855.69	1061.86^ab^ ± 34.07	1.87^b^ ± 0.18	14659.50^b^ ± 667.39	56.55^ab^ ± 4.02	1.12^a^ ± 0.03

*Note:* Values are given as mean ± SE of 6 animals unless otherwise stated. Values having different superscripts (a, b, c) in a column are statistically different from one another at a 5% level of significance. Values of CAT (Catalase) are expressed in μmol H_2_O_2_ decomposed/min/g of tissue. Values of SOD (Superoxide dismutase) are expressed in units/g of tissue. Activities of arylesterase (AE) expressed in U/mL. Acetylcholinesterase (AChE) activity expressed in nmol of thiol produced/min/mg of tissue. Values of malondialdehyde (MDA) are expressed in nmol of MDA formed/g/h. Values of advance oxidation protein product (AOPP) are expressed in μM of Chloramine‐T.

### 3.3 Genetic Expression Studies

**FIGURE 3:**
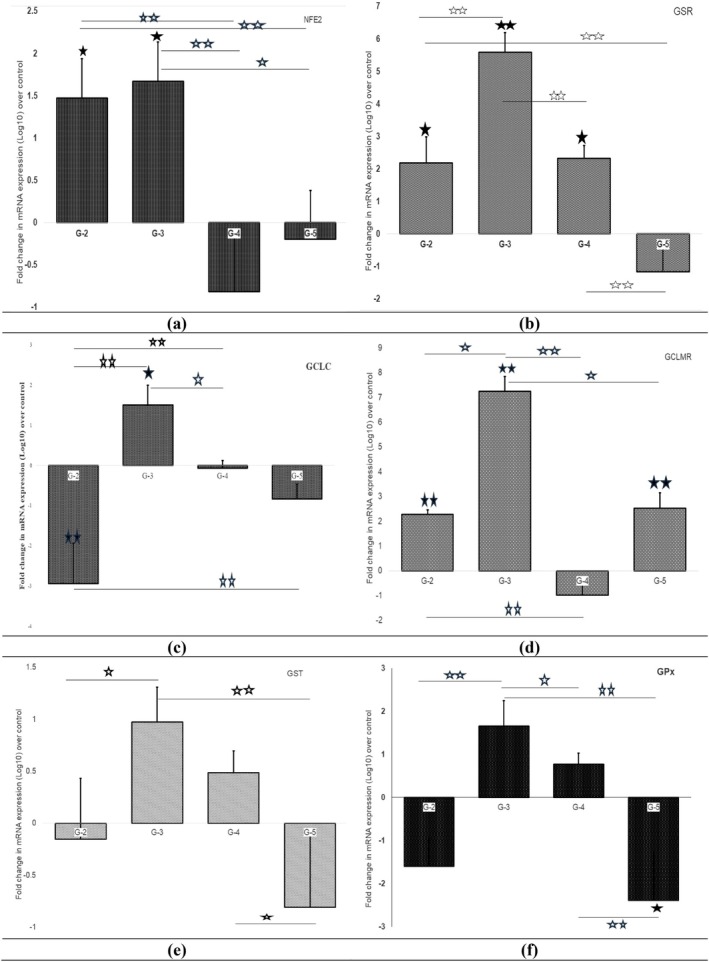
Fold change in mRNA expression level in nuclear factor E2 (Nrf_2_), glutathione reductase (GR), glutamate‐cysteine ligase catalytic subunit (GCLC), glutamate‐cysteine ligase modulatory subunit (GCLM), glutathione peroxidase (GPx), and glutathione s transferase (GST) in renal tissue of treatment groups over control (G‐2 = cisplatin; G‐3 = quercetin + cisplatin; G‐4 = catechin + cisplatin; G‐5 = genistein + cisplatin). Filled black star indicated values significantly differ from control and hallow star indicated value significantly differ between different groups [single star (p < 0.05) and double star (p < 0.001)].

The mRNA expression of NFE2, GR, GCLC, GCLM, GPx, and GST in different experimental groups is presented in Figure 3. There was significant upregulation in NFE2 (1.5‐fold), GSR (2‐fold), and GCLM (2‐fold) whereas GCLC was downregulated (about 3‐fold) while GPx and GST expression after cDDP toxicity did not alter in any significant measure from the corresponding levels in control rats.

**FIGURE 4:**
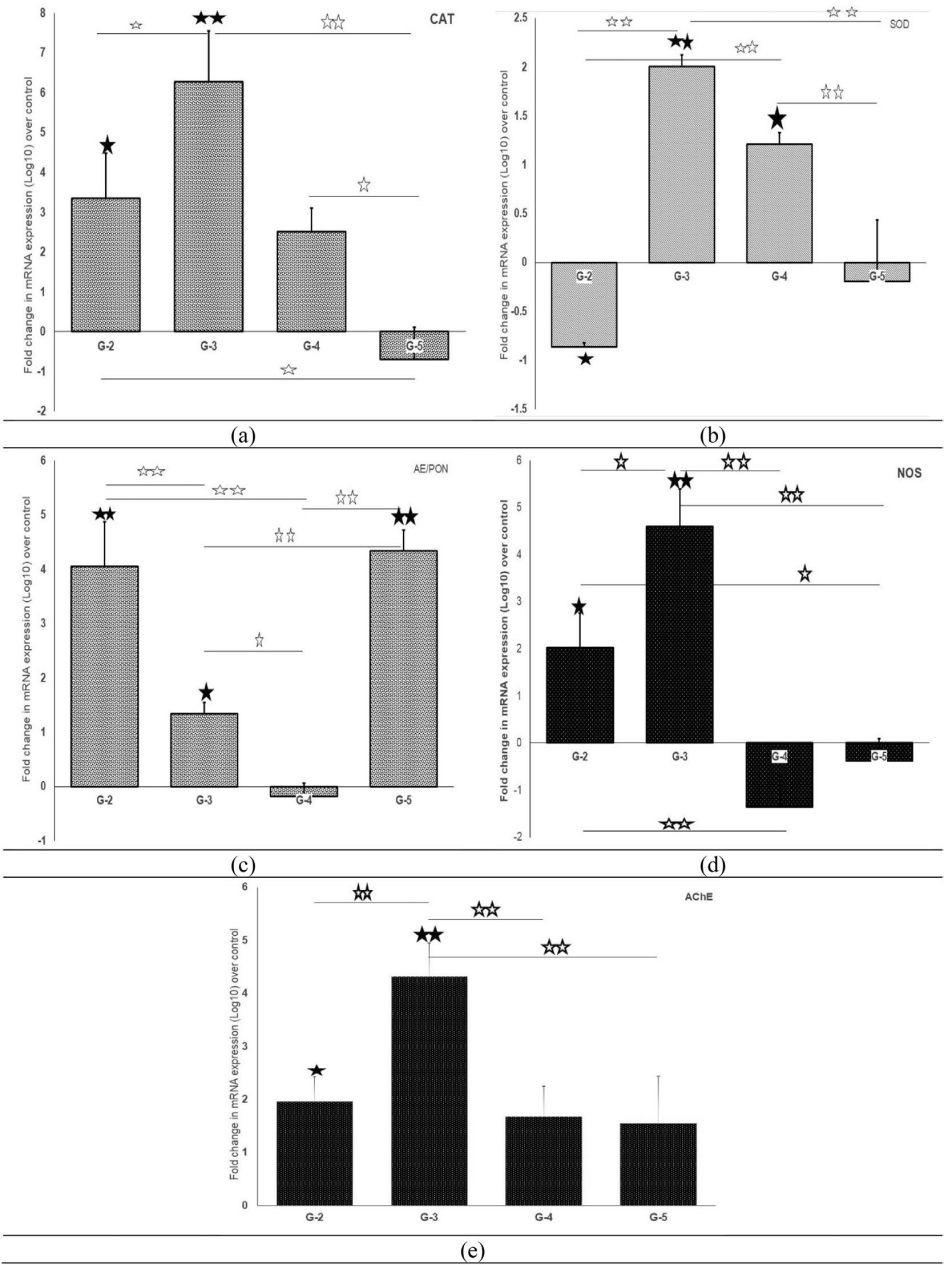
Fold change in the mRNA expression level of catalase (CAT), superoxide dismutase (SOD), arylesterase/PON, nitric oxide synthase (NOS), and acetylcholinesterase (AChE) enzymes in renal tissue of treatment groups over control (G‐2, cisplatin; G‐3, quercetin + cisplatin; G‐4, catechin + cisplatin; G‐5, genistein + cisplatin). Filled black star indicated values significantly differ from control and hallow star indicated value differ significantly between different groups [single star (p<0.05) and double star (p<0.001)].

Significant downregulation of NFE2 was recorded in catechin and genistein co‐treated rats as compared to cDDP alone, while no change in NFE2 expression was noticed after quercetin co‐treatment (Figure 3). For GSR, significant downregulation was only noticed in rats co‐exposed to genistein, whereas the quercetin group registered its significant upregulation, and in the catechin group, its expression levels remained unchanged when compared, respectively, to levels of GR in the cDDP only group. As far as the effect on GCLC expression was concerned, all three preventive agents significantly and equally upregulated mRNA expression in relation to its expression in the cDDP alone group. In contrast, significant downregulation of GCLM as compared to the cDDP alone treated group was seen only on catechin supplementation, while quercetin caused further upregulation of GCLM expression, and genistein made no changes to the expression of GCLC. As shown in Figure 4, cDDP treatment caused a significant upregulation of CAT (> 3‐fold), AE (4‐fold), NOS as well as AChE (2‐fold) whereas a significant downregulation of SOD expression occurred.

Significant reduction in expression of CAT with respect to cDDP alone group occurred only in rats given genistein; in contrast, quercetin significantly upregulated CAT, whereas catechin didn't alter expression levels of CAT when compared to the corresponding values in cDDP only treatment group. Quercetin as well as catechin, but not genistein, significantly reversed cDDP‐induced SOD downregulation and AE upregulation, whereas catechin as well as genistein treatment significantly reversed the upregulated NOS expression in cDDP‐intoxicated rats, wherein catechin treatment brought about a higher reduction in NOS expression levels. Catechin along with quercetin didn't alter AChE expression with respect to its levels in the cDDP alone group. In contrast, quercetin supplementation significantly upregulated expression when contrasted with AChE mRNA levels after cDDP intoxication.

### 3.4 Renal Histopathology

**FIGURE 5:**
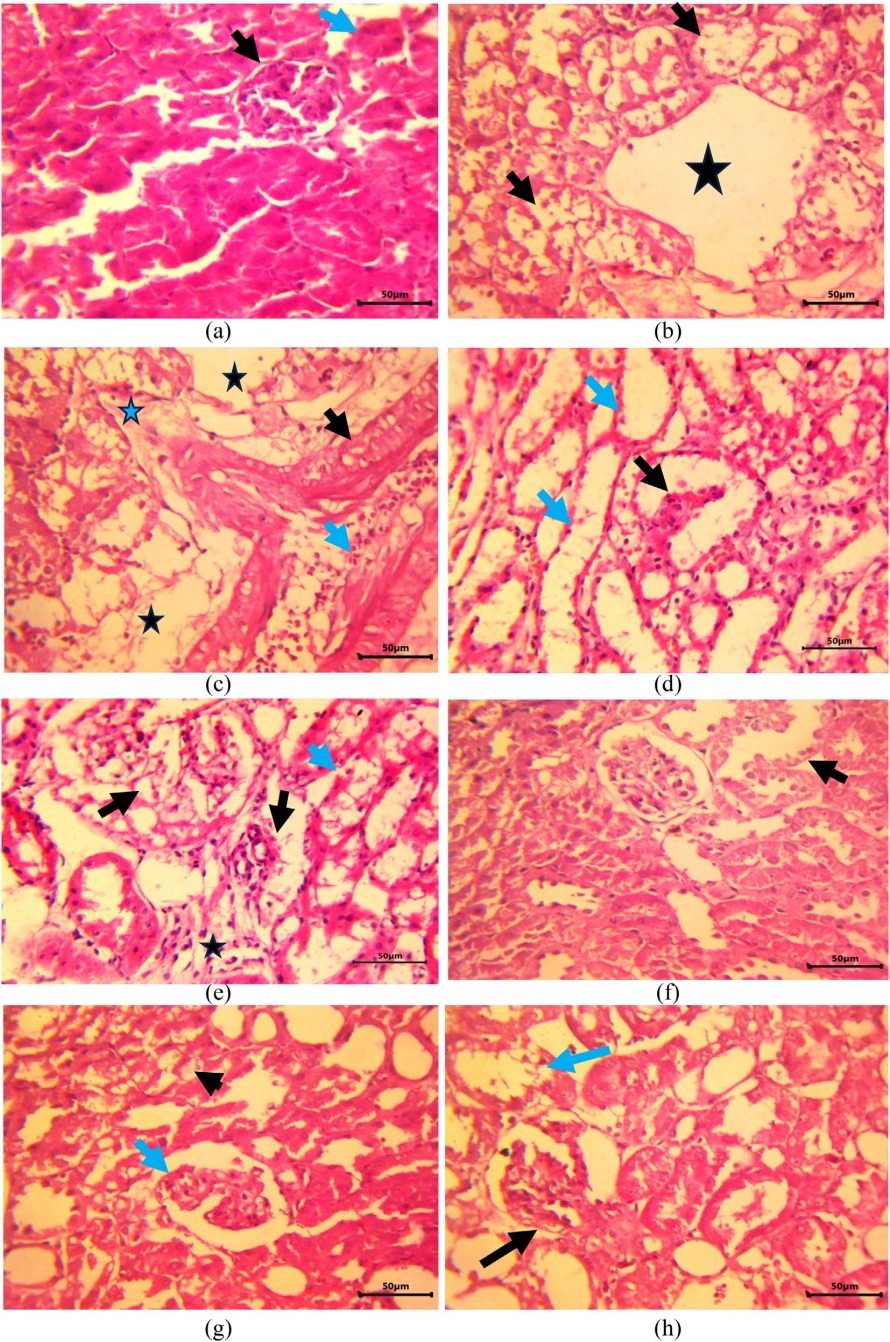
Histopathological alterations in renal tissue of Wistar rats (400×). (a) (Group I): The kidney section from the control group revealed normal glomerulus (black arrow) and healthy tubules (blue arrow). (b) (Group II): Tubular epithelium showing ballooning degeneration and necrosis (black arrow) and presence of fibrin and edema in the surrounding (star). (c) (Group II): Congested blood vessel (blue arrow) with the degenerated wall (black arrow) and edema and fibrin along with few inflammatory cells in perivascular space (blue star). (d) (Group II): Severely atrophic glomerular tufts (black arrow) with abnormal increase in the urinary space and the surrounding tubules showing complete denudation of lining epithelium. (e) (Group II): Disruption of Bowman's capsule with severe dilation, and destruction of capillary loops and eosinophilic debris in urinary space (black arrow). Presence of fibrin and inflammatory cells in the interstitium (star) and tubular vacuolar degeneration and tubulorhexis (blue arrow). (f) (Group III): Mild degeneration of renal epithelium (arrow) and glomerular tufts. (g) (Group IV): Mild tubular degeneration (black arrow) and atrophied glomerular tufts (blue arrow). (h) (Group V): Vacuolar degeneration (blue arrow) and glomerular degeneration (black arrow).

The control group had normal kidneys. Glomerular tufts enclosed within the Bowman's capsule did not display any pathological alteration (Figure 5a). Also, healthy renal tubules lined by cuboidal epithelium with centrally located nuclei were perched on the basement membrane. In rats administered cDDP, diffusely, the renal tubular epithelium showed striking ballooning degeneration. The cells were swollen and filled with coalescing vacuoles, and nuclei were often lost or pushed to the periphery (Figure 5b). Severely affected epithelium underwent necrosis with lysis of cells as well as the basement membrane. The wall of arterioles, which were also severely congested, revealed severe degenerative changes in the media. Consequently, edema and fibrin, along with a few inflammatory cells, were present in the surroundings (Figure 5c). There were pockets of edema and fibrin within the interstitium displacing the severely degenerated tubules (Figure 5b,c). Glomerular tufts also displayed divergent morphological alterations. In some rats, glomeruli diffusely were severely collapsed with abnormal increases in the urinary space, and the surrounding tubules either showed vacuolar degeneration or complete denudation of tubular lining epithelium (Figure 5d). However, in other rats, there was disruption of the Bowman's capsule alongside severe dilation, separation, or destruction of capillary loops, causing increased eosinophilic densities in the urinary space reflecting protein‐rich filtrate or necrotic debris. Periglomerular areas revealed the presence of fibrin and inflammatory cells in the interstitium and tubular epithelium manifesting severe vacuolar degeneration and tubulorhexis (Figure 5e). Quercetin was the most effective of the ameliorative agents used, and only mild degeneration of renal epithelium was seen in some foci (Figure 5f).

Rats given catechin also revealed only mild tubular degeneration, but focal degenerative changes were seen in the glomerulus (Figure 5g). Supplementation with genistein caused significant amelioration of pathological changes, with a marked reduction in the degree of degeneration of tubular epithelium as well as that of glomerular tufts, but was the least effective of amongst herbal drugs in conferring protection against the histopathological changes induced by cDDP (Figure 5h).

## 4 Discussion

Cisplatin is one of the most effective chemotherapeutic agents for a wide range of malignancies, particularly solid tumors, but its dose‐dependent toxicity is a prominent reason for the discontinuation of cDDP therapy that limits its clinical application (Galfetti et al. 2020). The acute kidney injury (AKI) occurring as a consequence of renal ischemia, oxidative stress, and inflammation is a well‐known side effect of the cDDP‐based antineoplastic regimen. The continuous cDDP use can result in chronic renal failure, necessitating the development of subsidiary therapies to neutralize its adverse effects on the kidney (Ozkok and Edelstein 2014). Antioxidants, whether acquired from exogenous sources or enzymes as active components of endogenous antioxidant machinery, are crucial in protection against oxidant‐induced tissue damage. Collateral damage to normal tissues in response to the usage of antineoplastic drugs can be balanced by using antioxidant supplements (Aldhahrani et al. 2025; Soliman et al. 2024). Scientific literature suggests the use of supplemental therapy in the form of medicinal plants with known pharmacological properties to ward off oxidative damage induced by chemotherapeutic drugs (Abdelhiee et al. 2025). Many herbal drugs have shown promising potential to alleviate renal injury induced by cDDP and can serve as treatment options (McSweeney et al. 2021). In the current paper, we sought to provide a further scientific explanation for the underlying mechanisms by which quercetin, catechin, and genistein can confer reno‐protection against cDDP‐induced nephrotoxicity.

The mammalian body has a robust endogenous antioxidant machinery that nullifies free radicals generated during normal cellular metabolic reactions. The antioxidant response mechanisms are primarily regulated by the induction of nuclear factor 2—antioxidant response element (Nrf2‐ARE) which is the key for activation of several enzymatic proteins, such as GCL, GPx, GR, GST, SOD, and CAT, as well as various non‐protein compounds viz. total thiols. Moreover, supplementation of various bioflavonoids can boost the body's inherent antioxidant defense mechanisms, as indicated by numerous experimental and epidemiological studies. A detailed explanation about the mechanisms of oxidative stress inflicted by cDDP in causing nephrotoxicity was provided by Zhou et al. (2022), who explicitly put forth depletion of endogenous enzymatic as well as non‐enzymatic antioxidants due to ROS generated in response to cDDP metabolism as the underlying cause. In the present study, high‐dose intraperitoneal cDDP administration significantly increased plasma levels of IL‐18 and GGT alongside renal biomarkers like BUN, CR, UA, and decreased levels of total plasma proteins and albumin, indicating the onset of AKI. Also, the transmembrane glycoprotein, KIM‐1, and cystatin‐C were elevated in response to damage to proximal tubular epithelium and a drop in glomerular filtration rate (Han et al. 2002; Murty et al. 2013). Previous reports have made similar observations and linked free radical‐induced oxidative stress to damage in renal tissue (Crona et al. 2017; Sharma et al. 2023; Singh, Singh, et al. 2020; Verma et al. 2018). In the present study, cDDP caused a reduction in levels or activities of GSH, TTH, GR, GPx, GST, GCLC, and GCLM in plasma and renal tissue. Biotransformation of cDDP occurs once it reaches renal epithelial cells, where it is conjugated with GSH, a reaction mediated by GST, leading to elevated levels of GSH conjugates in renal epithelial cells where they are further cleaved by GGT to cysteinyl‐glycine‐conjugates and ultimately nephrotoxic thiols. In a previous study, the GGT knockout animals did not develop cDDP‐induced AKI, although there were no differences in cDDP accumulation between the renal epithelium of wild‐type and GGT‐deficient mice (Hanigan et al. 2001). In the present study, increased plasma GGT levels were probably being assimilated in tubular epithelium to cause the accumulation of higher concentrations of cDDP‐derived toxic moieties. Increased utilization of the –SH group for scavenging intermediate metabolites may significantly overwhelm and reduce activities of GR, with the resultant reduced GSH replenishment, as indicated by reduced GCL activities in the present study. The levels of both subunits of GCL: GCLC and GCLM were significantly reduced, indicating cDDP toxicity glutted GCL activity. Further, the decreased levels of thiols are primarily responsible for the ensuing cascade of oxidative insults, as reduction in thiols impacts the activities of GPx, SOD, and GST, which require GSH as a substrate. Moreover, the decrease in activities of CAT, SOD, and AE in blood as well as in renal tissue of the cDDP exposed group added to the oxidative renal insult. Formation of DNA adducts, particularly 8‐OHdG, during biotransformation of cDDP forces cells into a cell‐cycle arrest (Valavanidis et al. 2009). The overall decline in the levels of antioxidants probably resulted in an unchecked accumulation of RONS in the renal tissue, causing damage to lipids, proteins, and nucleic acid. This was further corroborated by elevated 8‐OHdG levels and the results of histopathological studies. So, it can be inferred that the increased 8‐OHdG concentration in cDDP‐treated rats indicated oxidative damage to the DNA of renal epithelial cells owing to bioaccumulation and metabolism of cDDP, which probably contributed to cell death in renal tissue. Further, the presence of significant alterations in renal tissue viz. congestion, tubular degeneration alongside necrosis, and degeneration of blood vessel walls causing accumulation of edema fluid admixed with fibrin in the interstitium were also noticed. Moreover, glomerular tuft shrinkage, increased urinary space, and the presence of inflammatory cells in the interstitial spaces were prominent glomerular abnormalities. The cDDP intoxication caused a differential expression of most of the antioxidant genes studied, except GST (Verma et al. 2016). Notably, the mRNA expression results neither showed a consistent pattern nor showed complete alignment with respect to functional or activity data. For instance, cDDP administration led to a reduced expression of GPx and SOD, which correlated with their reduced concentrations in plasma, with both being reduced. However, increased mRNA expression but decreased activity was witnessed in the case of AChE, CAT, GR, and AE. On the contrary, GST's expression remained unchanged despite a reduction recorded in its activity. In the same vein, expression of Nrf_2_ and GCLM were upregulated, while that of GCLC was diminished by cDDP, in contrast to the observation of depletion in plasma levels of GCLC and GCLM subunits. It has also been noted in earlier works that altered expression does not always cause parallel chemical changes in toxicity studies viz. dysregulated gene expression induced by rotenone was not consistent with observable early developmental toxicity in zebrafish (Wilson et al. 2022). Notwithstanding the heterogeneity in our data, as is often the case in biological systems, it can be gleaned from our findings that levels of thiols and other antioxidants plummeted due to upstream disturbances in Nrf_2_, GCLM, and GCLC subunits. Several past studies have documented toxic renal damage after cDDP‐induced toxicity. Sandeep and Krishnan Nair (2010) found that administration of cDDP at pharmacologically relevant doses caused altered renal histomorphology alongside abnormal BUN and CR levels. At the same time, numerous scientific publications have emphasized the importance of naturally occurring antioxidants in curtailing these adverse effects (Zhou et al. 2022). Our findings have thrown light on the advantages of supporting cDDP therapy with quercetin, catechin, or genistein. Amelioration of kidney damage indices, such as BUN and CR, was comparable in all three preventive agents; however, UA was not improved by genistein treatment, while others exhibited significant improvement. The levels of KIM‐1 and cystatin‐C were completely restored by quercetin, and in contrast, genistein only improved KIM‐1, whereas catechin didn't affect either. Quercetin and catechin showed an overall stronger antioxidant activity than genistein, even though cDDP‐inflicted protein and membrane damage was restored by all three polyphenolic compounds. The DNA damage was completely masked by quercetin, less so with catechin, while its levels were not bettered by genistein at all.

The impact of ameliorative agents on mRNA expression also varied from their effect on the activities of many studied parameters in our research. Quercetin increased the expression of AChE, CAT, GPx, GR, and SOD and significantly improved the activities of AE, CAT, GPx, GSR, and SOD. Even though the expression of AChE was raised, this could not affect AChE activity levels; in contrast, quercetin reduced the expression of AE whereas AE activity was raised and normalized. Similarly, Tan et al. (2020) uncovered that quercetin could oppose cDDP‐induced toxicity in vitro and in vivo models of AKI, wherein serum levels of BUN and inflammatory cytokines were inhibited, leading to significant reversal of AKI.

Catechin also normalized the activities of AChE, CAT, GPx, and GR even though no change in expression was recorded for these parameters. On the contrary, expression as well as activity increased for SOD while both activity as well as expression were reduced in the case of AE. Similarly, epigallocatechin‐3‐gallate (EGCG) prevented CCDP nephrotoxicity in mice by reducing BUN as well as CR and blocking apoptosis (Zou et al. 2014). Malik et al. (2016) provided the mechanistic details of renal protection offered by epicatechin gallate against cDDP nephrotoxicity, which included suppression of inflammation as well as reduction of oxidative stress by restoration of SOD, CAT, and decreasing MDA besides blocking apoptosis. Oxidative stress markers (SOD, GSH, CAT, and GPx) and biochemical markers of kidney injury due to cDDP‐induced AKI in rats were markedly attenuated by gallic acid pre‐treatment (Eslamifar et al. 2021). Catechin improved levels of SOD and GSH but not MDA after concurrent CCDP treatment in HEK293 cells although it was not effective in increasing their survivability (Wang et al. 2017). Epigallocatechin gallate in combination with coenzyme Q10 mitigated cDDP‐mediated nephrotoxicity through alleviation of oxidative as well as nitrosative stress, inflammation, apoptosis, and histomorphological damage and thereby caused enhancement in cDDP efficacy as an antineoplastic drug (Fatima et al. 2016).

Genistein administration did not alter the expressions of AChE, GPx, SOD, and AE but reduced the expression of CAT and GR. However, following its supplementation, activities of AChE, GPx, SOD, and AE were normalized, but CAT levels could not be rescued. A large body of existing literature suggests isoflavones such as genistein must be harnessed as supporting therapy to sabotage anticancer chemotherapeutics mediated severe side effects like nephrotoxicity and to augment their antineoplastic potential (Sahin et al. 2019). Available literature also points to the presence of formidable anti‐inflammatory properties in genistein exerted via suppression of oxidative stress (Goh et al. 2022). Genistein restored intestinal ALP levels and improved diarrhea in rats subjected to irinotecan hydrochloride toxicity (Yokooji et al. 2012).

Even while quercetin didn't alter Nrf_2_, catechin and genistein brought about a significant depreciation in its levels. All three herbal agents could significantly uplift GCLC expression while only catechin was useful in significant downregulation of GCLM. Strikingly, GCLC and GCLM activities showed complete amelioration upon supplementation with quercetin and catechin while genistein was not effective in the rectification of GCLM. Earlier, researchers have shown that quercetin inhibited overexpression of SOD, CAT, and Nrf_2_ engineered via redox‐mediated mechanisms and reversed cDDP resistance in cancer cells while it enhanced the expression of these enzymes in normal cells (Hasan et al. 2022). Shim et al. (2024) demonstrated that catechin hydrate could effectively block apoptosis, oxidative stress, inflammatory response, and Nrf_2_ expression in male germ cells (GC‐1 spg) undergoing cDDP treatment. Also, dihydromyricetin showed formidable potency in alleviating levels of BUN, CRE, and KIM‐1, upregulation of expression of CAT, Nrf2, GCLC, and GCLM; besides, it regressed inflammatory response and damage to renal parenchyma by modulating the formation of inflammazone (Xu et al. 2023). Taxifolin leveled the raised BUN, CR, and MDA levels and upregulated Nrf2 in response to cDDP (Alanezi et al. 2022). Overall, our data show that quercetin, catechin, and genistein plausibly salvaged endogenous thiols under siege of oxidative fatigue spurred by cDDP toxicity by upregulation of the Nrf_2_ pathway and its downstream players, particularly GCLC and GCLM, which plausibly bolstered the endogenous antioxidant prowess, particularly the thiols.

All three medicinal ingredients in our study markedly blocked histopathological changes brought about by cDDP, but mild degeneration in tubular epithelium and glomerulus was still observed. Quercetin conferred the highest protection, followed by catechin and genistein in that order. Shi et al. (2022) formulated a Chinese herb mixture with anti‐inflammatory properties, which effectively prevented CCDP‐induced adverse side effects on the kidney along with augmenting cDDP chemosensitivity. Similarly, traditional Korean polyherbal formulations enhanced cell viability, SOD, CAT activities, and free radical scavenging but reduced apoptosis in renal epithelium to attenuate AKI inflicted by cDDP (Dachuri et al. 2020). Chinese herbal mixture was proposed as a possible adjuvant treatment to decrease cDDP nephrotoxicity (Guo et al. 2018) as it could attenuate BUN, congestion, and renal tubular injury mediated via reduced production of TLR‐4, NF‐κB, and TNF‐a. Afsar et al. (2021) recommended *A. hydaspica* extract as a probable treatment option to counter AKI induced by CCDP as it amended concentrations of renal biomarkers, maintained the integrity of antioxidant machinery, and reduced the expression as well as plasma levels of proinflammatory cytokines. Flavanol Kaempferide (derived from *Kaempferia galanga* ) ameliorated renal tissue damage and dysfunction induced by cDDP in mice, as evidenced by downregulation of SOD, KIM‐1, effective strengthening of antioxidant functioning, and preservation of cell viability, plus a significant reduction in tubular damage and inflammation (Shao et al. 2023). Shi et al. (2024) used in vitro as well as in vivo models to emphasize the clinical importance of quercetin in counteracting cDDP‐induced acute kidney damage via suppressing metal‐dependent cell death mechanisms viz. ferroptosis by attenuating MDA and boosting GSH levels and inhibiting cuproptosis by reducing levels of copper ion, pyruvate, and HSP70.

These results concur with our findings of the turnaround seen in cDDP‐induced impairment and exhaustion in activities of thiols and other antioxidant enzymes owing to the effect of quercetin, catechin, or genistein to ultimately reduce oxidative stress, which got manifested in the form of improvement in renal functions as well as histomorphology. In a nutshell, the results of this study show herbal extracts enriched with flavanols viz. quercetin, catechin, and genistein hold immense promise to counter cDDP‐induced AKI. However, all our findings taken together indicate that quercetin showed the maximum ameliorative potential among the three herbal agents used against cDDP‐induced renal toxicity in the study. Also, catechin conferred greater preventive abilities against oxidative damage than genistein against CDDP‐induced nephrotoxicity.

## 5 Conclusions

Intraperitoneal administration of cDDP adversely impacted the biochemical indices of renal health and activities of antioxidant biomarkers in blood and kidney in an experimental model. Besides a noticeable alteration in the expression of several parameters of the antioxidant system, the histoarchitecture of the kidney was also observed, indicative of renal damage. Quercetin, catechin, and genistein showed potential as prospective remedial agents to lower cDDP nephrotoxicity in rats when individually used as additives alongside cDDP. However, quercetin showed maximum protective potential among all three phytochemicals used. Our study contributed towards the widening of existing knowledge on phytochemicals that can be used as supplemental therapy to diminish the impact of cDDP toxicity on the kidney.

## Author Contributions

**Pawan K. Verma:** conceptualization (equal), writing – original draft (equal), writing – review and editing (equal). **Priyanka Sharma:** resources (equal), software (equal). **Sheen Tukra:** conceptualization (equal), data curation (equal), resources (equal), writing – original draft (equal), writing – review and editing (equal). **Bhawani Singh:** validation (equal), writing – original draft (equal), writing – review and editing (equal). **Shilpa Sood:** data curation (equal), formal analysis (equal), writing – original draft (equal). **Nrip K. Pankaj:** conceptualization (equal), formal analysis (equal), writing – original draft (equal), writing – review and editing (equal). **Biswajit Brahma:** formal analysis (equal), writing – original draft (equal), writing – review and editing (equal). **Gholamreza Abdi:** conceptualization (equal), formal analysis (equal), methodology (equal), resources (equal), software (equal), supervision (equal), validation (equal), writing – original draft (equal), writing – review and editing (equal). **Zuhaib F. Bhat:** software (equal), supervision (equal), validation (equal), writing – review and editing (equal).

## Consent

The authors have nothing to report.

## Conflicts of Interest

The authors declare no conflicts of interest.

## Data Availability

The datasets used and/or analyzed in the present study are available from the corresponding author on reasonable request.
